# Occupational risk factors for hip osteoarthritis are associated with early hip structural abnormalities: a 3.0 T magnetic resonance imaging study of community-based adults

**DOI:** 10.1186/s13075-015-0535-3

**Published:** 2015-01-28

**Authors:** Andrew J Teichtahl, Sam Smith, Yuanyuan Wang, Anita E Wluka, Richard O’Sullivan, Graham G Giles, Flavia M Cicuttini

**Affiliations:** Department of Epidemiology and Preventive Medicine, School of Public Health and Preventive Medicine, Monash University, Alfred Hospital, 99 Commercial Road, Melbourne, VIC 3004 Australia; Baker IDI Heart and Diabetes Institute, 75 Commercial Road, Melbourne, VIC 3004 Australia; MRI Department, Healthcare Imaging Services, Epworth Hospital, 89 Bridge Road, Richmond, VIC 3121 Australia; Department of Medicine, Central Clinical School, Monash University, 55 Commercial Road, Melbourne, VIC 3004 Australia; Cancer Epidemiology Centre, Cancer Council Victoria, 89 Bridge Road, Melbourne, VIC 3004 Australia; Centre for Epidemiology and Biostatistics, Melbourne School of Population and Global Health, The University of Melbourne, 207 Bouverie Street, Carlton, VIC 3053 Australia

## Abstract

**Introduction:**

Occupational exposure to heavy lifting and stair climbing are associated with radiographic hip osteoarthritis (OA). This study examined whether these activities are associated with early structural hip joint changes in a community-based population.

**Methods:**

In total, 198 community-based people with no history of hip disease, including OA, had 3.0 T-magnetic resonance imaging (MRI) to assess hip cartilage volume, defects and bone marrow lesions (BMLs). Recall of occupational exposure to heavy lifting and stair climbing aged 18 to 30 years and in the previous 10 years were collected. A persistence score was defined as exposure at neither time point (0), at one time point (1) or at both time points (2).

**Results:**

Exposure to heavy lifting when aged 18 to 30 years was associated with BMLs of the central superolateral femoroacetabular region (odds ratio (OR) 3.9, 95% confidence interval (CI) 1.6 to 9.8, *P* <0.01), with persistence score associated with cartilage defects in the central superolateral region of the femoral head (OR 1.6, 95% CI 1.0 to 2.5, *P* = 0.04). Exposure to stair climbing aged 18 to 30 years and persistence score were associated with an increased risk of cartilage defects in the central superolateral femoral head and BMLs in the central superolateral and posterior femoroacetabular regions (OR range 2.1 to 3.2, all *P* ≤0.03).

**Conclusions:**

Occupational exposure to heavy lifting and stair climbing are associated with hip structural abnormalities. If confirmed by longitudinal data, such associations may explain how occupational activities affect the hip joint and may identify new targets for the prevention of hip OA.

## Introduction

Hip osteoarthritis (OA) is a common and disabling condition that in its most severe form requires costly joint replacement surgery. There is evidence for both genetic [1] and environmental factors, including occupational activity [2], having a role in its pathogenesis.

Of the occupational activities examined, a systematic review found that 12 of 14 studies demonstrated a significantly increased risk of hip OA for people exposed to heavy lifting [2]. Only five previous studies have investigated the association between stair climbing and hip OA, with a positive association reported by all, despite not always reaching statistical significance [3-7]. A systematic review concluded that future research should focus on longer follow-up time, dose responses and utilise newer outcome methods for assessing the joint, such as magnetic resonance imaging (MRI) [2]. MRI enables a non-invasive assessment of the structural features of early hip OA. For instance, using MRI, a 13% mean reduction in femoral head cartilage volume is demonstrable before any evidence of radiographic joint space narrowing [8]. Other structural changes determined from MRI, such as the presence of hip cartilage defects and bone marrow lesions (BMLs) have also been associated with self-reported hip pain, disability and radiographic OA [9-11]. Such advances in imaging have enabled joint diseases such as OA to be examined in the early pre-radiographic stage to determine whether variables such as occupational activity are associated with structural abnormalities in the hip joint.

Our aim in this study was to examine the associations between occupational heavy lifting and stair climbing over a working lifetime and structural abnormalities of the hip joint assessed from MRI in a community-based sample of individuals with no diagnosed hip OA.

## Methods

### Participants

Participants were recruited between 2009 and 2010 from the Melbourne Collaborative Cohort Study (MCCS), a prospective cohort study of 41,514 residents of Melbourne, Australia, aged 27 to 75 years (99.3% aged 40 to 69 years) at MCCS inception (1990 to 1994) [12]. Participants were recruited via Electoral Rolls (registration to vote is compulsory for Australian adults), advertisements, and community announcements in the local media (for example, television, radio, newspapers), between 1990 and 1994. Participants were eligible for the current study if they did not meet any of the following exclusion criteria: a medical or allied health professional-made diagnosis of hip OA, significant hip pain lasting for >24 hours in the last 5 years (requiring medical assessment, intervention or non-weight bearing); surgery (including arthroscopy); a malignancy; a history of any form of arthritis diagnosed by a medical practitioner; or a contraindication to MRI including pacemaker, metal sutures, presence of shrapnel or iron filings in the eye, or claustrophobia. To ensure that we captured a relatively pain-free population, we assessed pain using the Western Ontario and McMasters Universities Arthritis Index (WOMAC) pain subscale.

The study was approved by The Cancer Council Victoria’s Human Research Ethics Committee and Monash University Human Research Ethics Committees. All participants gave written informed consent.

### Anthropometric data

Anthropometric data were collected at the time of MRI assessment. Height was measured using a stadiometer and weight using electronic scales. Body mass index (BMI) (weight/height^2^, kg/m^2^) was calculated.

### Occupational activity

Occupational activity data were collected using a questionnaire at the visit of hip MRI. The participants were asked to recall their occupational exposure to heavy lifting and stair climbing in the past 10 years and between the ages of 18 and 30 years: whether they lifted weights greater than 10 kg at least 10 times in an average working week in a job they held for at least one year, and whether they climbed more than 30 flights of stairs in their average working day in a job they held for at least one year.

The persistence of exposure to each occupational activity (heavy lifting or stair climbing) was assessed by devising the following score for each activity: 0 - no exposure at either time point, 1 - exposure at either, but not both time points and 2 - exposure at both time points. This score was hereafter termed ‘persistence’.

### Recreational and domestic activities

Recreational and domestic activities were assessed using the Physical Activity Scale for the Elderly (PASE), a reliable and valid tool to assess physical activity in epidemiologic studies of older people [13]. Vigorous physical activity in the 7 days preceding MRI was assessed by asking whether a participant had performed at least 20 consecutive minutes of vigorous exercise, severe enough to cause shortness of breath or sweating, with examples such as swimming, tennis, netball, athletics and running provided. Similarly, heavy domestic chores in the 7 days preceding MRI were assessed by asking whether a participant had performed heavy housework chores, such as vacuuming, scrubbing floors, washing windows or carrying wood.

### MRI measurements

Each participant without a diagnosis of hip OA had an MRI performed on their dominant hip, defined by the leg used to kick a ball (89% right sided) in 2009 and 2010. MRI was performed at two locations (Epworth Hospital Richmond and Box Hill (n = 132. 67.3%), VIC, Australia). At each site, hips were imaged on a 3.0-T whole-body magnetic resonance unit (Siemens, Verio, Siemens Medical, Erlangen, Germany) using a phased array flex coil. Sagittal images were obtained using a T_2_-weighted fat-suppressed three-dimensional gradient-recalled acquisition sequence in the steady state (repetition time 14.45 msec, echo time 5.17 msec; flip angle 25°, slice thickness 1.5 mm, field of view 16 cm, pixel matrix 320 × 320, acquisition time 7 minutes 47 seconds, and 1 acquisition). Coronal images were obtained using a fat saturation, proton density, spin echo acquisition sequence (repetition time 3,400 msec, echo time 64 msec, flip angle 90°, slice thickness 3 mm, field of view 16 cm, pixel matrix 256 × 256, acquisition time 5 min 26 sec, and 1 acquisition). A musculoskeletal radiologist with over 15 years’ experience using structural outcomes determined by MRI in epidemiological studies supervised and independently monitored measurements. One observer, trained by the radiologist was responsible for measuring one structural outcome (for example cartilage volume, cartilage defects or BMLs). Each observer was also required to assess their designated structural measure in duplicate, at least one week apart and blinded to their previous assessment and characteristics of the participants.

Femoral head cartilage volume was measured from T_2_-weighted sagittal images using the software Osiris (version 4.19; Geneva University Hospital, Geneva, Switzerland) as previously described [8]. The image data were transferred to the workstation, and an isotropic voxel size was then obtained by a trilinear interpolation routine. The volume of the femoral head cartilage was isolated from the total volume by manually drawing disarticulation contours around the cartilage boundaries on each image section. These data were then resampled by bilinear and cubic interpolation for the final three-dimensional rendering. The volume of the femoral head cartilage was determined by summing all the pertinent voxels within the resultant binary volume. Femoral head cartilage volume was measured in duplicate with at least a 1-week interval by one trained observer. The coefficient of variation (CV) was 2.5% [8]. The intraobserver reproducibility (assessed by intraclass correlation coefficient, ICC) was 0.99.

**Figure 1:**
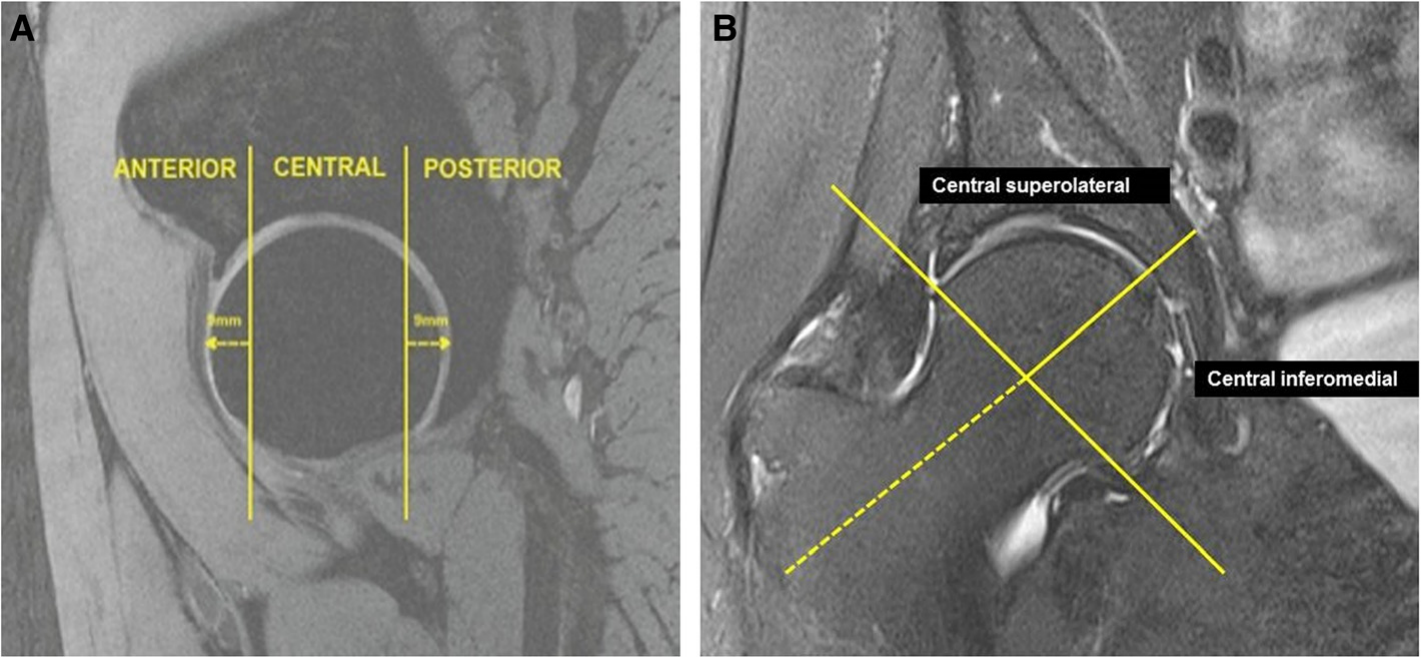
**Regional zones of the hip joint. (A)** Sagittal image depicting the anterior, central and posterior regions; **(B)** coronal image depicting the central superolateral and inferomedial regions.

The femoral head was divided into three regions: central, anterior and posterior to assess cartilage defects and BMLs. The anterior and posterior regions were assessed in the sagittal plane and corresponded to the first and last three coronal slices (9 mm) (Figure 1A). The area in between the anterior and posterior region was termed the central region. The division of anterior, central and posterior regions was adapted from methods used in previously published works [10,11]. The central region was further subdivided in the coronal plane (Figure 1B). The intersection of the axis of the femoral head and neck was considered to be the midpoint of the region, with the axis of the femoral neck used to demarcate the central superolateral from the central inferomedial region. Femoral head cartilage defects and BMLs were assessed from proton density coronal images and confirmed on sagittal imaging for the central region, and from the sagittal imaging for the anterior and posterior regions. The presence of cartilage defects was defined as loss of cartilage thickness of more than 50% which was shown on at least two consecutive slices. The presence of a BML was defined if it appeared on two or more consecutive slices. One trained observer, who was blinded to participant’s characteristics, assessed the presence of cartilage defects and BMLs for each participant in duplicate, at least one week apart. The intraobserver reproducibility (kappa) was 0.82 for cartilage defects and 0.93 for BMLs.

The sagittal image closest to the centre of the femoral head was used to measure the femoral head bone area. It was measured by drawing contours around the femoral head bone, and area calculated automatically by the Osiris program as an indicator of bone size. Femoral head bone area was measured by one trained observer with 50 random cross-checks performed by a second observer. The CV was 1.1% [8]. The inter-observer reproducibility (ICC) was 0.99.

### Statistical analyses

The outcome measures were the prevalence of hip cartilage defects and BMLs, as well as femoral head cartilage volume. Binary logistic regression was used to determine the associations between occupational activity between the ages of 18 and 30 years and persistence score with the prevalence of cartilage defects and BMLs. Linear regression analyses were used to determine the associations between occupational activity and femoral head cartilage volume. A *P* value of less than 0.05 (two-tailed) was regarded as statistically significant. All analyses were performed using SPSS statistical package (standard version 20.0 SPSS, Chicago, IL, USA).

## Results

One hundred and ninety-eight subjects who had MRI provided a response when asked about their occupational activity when aged 18 to 30 years. One hundred and sixty-three subjects who had MRI provided a response when asked about their occupational activity in the previous 10 years. The discrepancy between the number of people who provided a response to occupational exposure when aged 18 to 30 years and those in the previous 10 years was attributable to retirement in the intervening period. Relative to those people still working, retired subjects tended to be older (74.7 versus 65.6 years *P* <0.0001) and less likely to be males (47.2% versus 24.7% males, *P* = 0.02). Subject characteristics are shown in Table 1. The mean age of the cohort was 67.1 (±7.7) years, and 43.4% were males. The median of the total WOMAC pain score (out of 500) was 20. Forty-six (28.7%), 52 (32.5%) and 62 (38.8%) people had performed occupational heavy lifting at no, one or both time points respectively. One hundred and twenty-two (62.2%), 28 (14.3%) and 13 (6.6%) people had performed occupational stair climbing at no, one or both time points respectively. There was a strong correlation between the exposure to occupational activity aged 18 to 30 years and the persistence score for both heavy lifting (r = 0.81, *P* <0.001) and stair climbing (r = 0.85, *P* <0.0001). Most (76.2%) of the cohort had performed heavy domestic chores in the previous 7 days, while a smaller number (23.4%) had performed vigorous physical activity in the 7 days preceding MRI.

**Table 1:** **Characteristics of study participants (N = 198)**

**At time of MRI**	
Age (years)	67.1 (7.7)
Gender (n, % male)	85 (43.4)
BMI (kg m^−2^)	27.2 (4.6)
Heavy domestic chores in previous 7 days, n (%)	163 (76.2)
Vigorous physical activity in previous 7 days, n (%)	50 (23.4)
WOMAC pain score, median	20
Femoral head cartilage volume (mm^3^)	3259 (810)
Femoral head bone area (mm^2^)	1615 (266)
**Femoral head cartilage defects, n (%)**	
*Anterior*	9 (4.5)
*Central superolateral*	72 (36.7)
*Central inferomedial*	104 (53.1)
*Posterior*	36 (18.2)
**Femoroacetabular BMLs, n (%)**	
*Anterior*	34 (17.2)
*Central superolateral*	40 (20.4)
*Central inferomedial*	12 (6.1)
*Posterior*	22 (11.1)
**Occupational heavy lifting n (%)**	
**Aged 18 to 30 years**	114 (59.7)
**Previous 10 years**	76 (38.8)
**Persistence score**	
*No time point*	46 (28.7)
*One time point*	52 (32.5)
*Both time points*	62 (38.8)
**Occupational stair climbing n (%)**	
**Aged 18 to 30 years**	37 (18.9)
**Previous 10 years**	23 (11.7)
**Persistence score**	
*No time point*	122 (62.2)
*One time point*	28 (14.3)
*Both time points*	13 (6.6)

Mean (standard deviation) unless otherwise stated. MRI, magnetic resonance imaging; BMI, body mass index; WOMAC, Western Ontario and McMasters Universities Arthritis Index; BMLs, bone marrow lesions.

Table 2 demonstrates the associations between heavy lifting and structural changes at the hip. Exposure to heavy lifting aged 18 to 30 years was significantly associated with the risk of BMLs in the central superolateral region of the femoroacetabulum after adjustment for age, gender, BMI, femoral head cartilage volume, MRI centre, vigorous physical activity and heavy domestic chores in the past 7 days (odds ratio (OR) 3.9, 95% confidence interval (CI) 1.6 to 9.8, *P* <0.01). The persistence score for heavy lifting was significantly associated with the risk of cartilage defects in the central superolateral region of the femoral head (OR 1.6, 95% CI 1.0 to 2.5, *P* = 0.04) after adjusting for age, gender, BMI, femoral head cartilage volume, MRI centre, vigorous physical activity and heavy domestic chores in the past 7 days.

**Table 2:** **Associations between occupational heavy lifting and hip structural changes**

	**Univariate** **β** **or odds ratio (95% CI)**	***P***	**Multivariate** **β** **or odds ratio (95% CI) Model 1**	***P***	**Multivariate** **β** **or odds ratio (95% CI)** ^**4**^ **Model 2**	***P***
**Heavy lifting aged 18 to 30 years (yes versus no)**						
Femoral head cartilage volume^1^	327 (97, 556)	<0.01	18 (−127, 164)	0.80	15 (−132, 162)	0.84
**Femoral head cartilage defects** ^2^						
Anterior	5.7 (0.7, 46.3)	0.11	5.2 (0.6, 44.7)	0.13	5.0 (0.6, 43.1)	0.14
Central superolateral	1.8 (0.8, 3.3)	0.06	1.8 (0.9, 3.4)	0.08	1.8 (0.9, 3.4)	0.09
Central inferomedial	1.5 (0.8, 2.6)	0.17	1.3 (0.7, 2.4)	0.35	1.3 (0.7, 2.4)	0.37
Posterior	1.7 (0.8, 3.5)	0.19	1.8 (0.8, 4.0)	0.15	1.8 (0.8, 4.1)	0.14
**Femoroacetabular BMLs** ^3^						
Anterior	1.0 (0.5, 2.1)	0.93	1.2 (0.6, 2.6)	0.63	1.2 (0.5, 2.6)	0.67
Central superolateral	4.3 (1.8, 10.4)	<0.01	3.8 (1.5, 9.2)	<0.01	3.9 (1.6, 9.8)	<0.01
Central inferomedial	4.0 (0.9, 18.4)	0.08	4.4 (0.9, 21.6)	0.07	5.0 (1.0, 26.2)	0.06
Posterior	2.0 (0.8, 5.4)	0.15	2.3 (0.8, 6.5)	0.10	2.5 (0.9, 7.0)	0.08
**Persistence score for heavy lifting (grades 1 to 3)**						
Femoral head cartilage volume^1^	189 (33, 345)	0.02	48 (−50, 146)	0.33	49 (−49, 148)	0.32
**Femoral head cartilage defects** ^2^						
Anterior	2.4 (0.8, 7.7)	0.13	3.1 (0.8, 11.0)	0.10	3.1 (0.8, 12.0)	0.11
Central superolateral	1.6 (1.1, 2.5)	0.02	1.6 (1.0, 2.5)	0.04	1.6 (1.0, 2.5)	0.04
Central inferomedial	1.1 (0.8, 1.6)	0.55	1.0 (0.7, 1.5)	0.93	1.0 (0.7, 1.6)	0.89
Posterior	1.2 (0.7, 2.0)	0.44	1.2 (0.7, 2.1)	0.48	1.2 (0.7, 2.1)	0.44
**Femoroacetabular BMLs** ^3^						
Anterior	0.8 (0.5, 1.3)	0.34	0.8 (0.5, 1.3)	0.33	0.8 (0.5, 1.3)	0.32
Central superolateral	1.6 (1.0, 2.6)	0.05	1.5 (0.9, 2.5)	0.11	1.5 (0.9, 2.6)	0.09
Central inferomedial	1.9 (0.8, 4.4)	0.14	1.7 (0.7, 4.1)	0.27	1.7 (0.7, 4.3)	0.25
Posterior	1.9 (1.0, 3.8)	0.06	1.9 (1.0, 3.8)	0.07	2.0 (1.0, 4.1)	0.05

^1^Regression coefficient (β) (95% CI) where for every one unit increase in the exposure (occupational heavy lifting), there is an associated increase (+) or decrease (−) in femoral head cartilage volume (mm^3^) adjusted for age, gender, BMI, femoral head bone area and MRI centre in Model 1; ^2^odds ratio (OR) where for every one unit increase in the exposure (occupational heavy lifting), there is an associated increased risk (value >1) or decreased (value <1) risk for cartilage defects, adjusted for age, gender, BMI, femoral head cartilage volume and MRI centre in Model 1; ^3^odds ratio (OR) where for every one unit increase in the exposure (occupational heavy lifting), there is an associated increased risk (value >1) or decreased (value <1) risk for BMLs adjusted for age, gender, BMI and MRI centre in Model 1; ^4^Model 2: vigorous physical activity in past 7 days and heavy domestic chores in past 7 days added to Model 1 multivariate equation. CI, confidence interval; BMLs, bone marrow lesions; BMI, body mass index; MRI, magnetic resonance imaging.

Table 3 describes the associations between stair climbing and structural changes at the hip. Exposure to stair climbing when aged 18 to 30 years of age was significantly associated with an increased risk of cartilage defects in the central superolateral region of the femoral head (OR 3.2, 95% CI 1.4 to 7.3, *P* <0.01) as well as BMLs in the central superolateral (OR 2.5, 95% CI 1.1 to 5.7, *P* = 0.03) and posterior (OR 3.2, 95% CI 1.1 to 8.9, *P* = 0.03) regions of the femoroacetabulum. The persistence score for stair climbing was significantly associated with an increased risk of both cartilage defects in the central superolateral region of the femoral head (OR 2.4, 95% CI 1.3 to 4.3, *P* <0.01) and BMLs in the central superolateral (OR 2.1, 95% CI 1.2 to 3.8, *P* = 0.01) and posterior (OR 2.3, 95% CI 1.1 to 4.9, *P* = 0.03) regions of the femoroacetabulum.

**Table 3:** **Associations between occupational stair climbing and hip structural changes**

	**Univariate** **β** **or odds ratio (95% CI)**	***P***	**Multivariate** **β** **or odds ratio (95% CI)**	***P***	**Multivariate** **β** **or odds ratio (95% CI)** ^**4**^	***P***
**Stair climbing aged 18 to 30 years (yes versus no)**						
Femoral head cartilage volume^1^	502 (217, 786)	<0.01	30 (−151, 211)	0.74	29 (−155, 213)	0.76
**Femoral head cartilage defects** ^2^						
Anterior	3.6 (0.9, 14.3)	0.06	5.3 (1.0, 27.5)	0.05	4.8 (0.9, 25.4)	0.06
Central superolateral	2.4 (1.2, 5.0)	0.02	3.1 (1.4, 7.0)	<0.01	3.2 (1.4, 7.3)	<0.01
Central inferomedial	0.7 (0.3, 1.4)	0.32	0.6 (0.3, 1.3)	0.20	0.6 (0.3, 1.3)	0.17
Posterior	1.0 (0.4, 2.4)	0.95	1.0 (0.4, 2.7)	0.97	1.0 (0.4, 2.6)	0.98
**Femoroacetabular BMLs** ^3^						
Anterior	0.9 (0.3, 2.3)	0.79	1.2 (0.4, 3.3)	0.73	1.1 (0.4, 3.0)	0.87
Central superolateral	3.1 (1.4, 6.8)	<0.01	2.6 (1.2, 5.9)	0.02	2.5 (1.1, 5.7)	0.03
Central inferomedial	0.4 (0.0, 3.0)	0.35	0.5 (0.1, 4.1)	0.50	0.4 (0.1, 3.9)	0.46
Posterior	2.8 (1.1, 7.2)	0.04	3.2 (1.2, 8.9)	0.03	3.2 (1.1, 8.9)	0.03
**Persistence score for stair climbing (grades 1 to 3)**						
Femoral head cartilage volume^1^	308 (104, 511)	<0.01	−53 (−182, 75)	0.41	−56 (−185, 73)	0.39
**Femoral head cartilage defects** ^2^						
Anterior	0.6 (0.1, 4.1)	0.63	0.6 (0.1, 4.4)	0.64	0.6 (0.1, 4.2)	0.58
Central superolateral	1.9 (1.1, 3.2)	0.01	2.3 (1.3, 4.0)	<0.01	2.4 (1.3, 4.3)	<0.01
Central inferomedial	0.8 (0.5, 1.3)	0.29	0.7 (0.4, 1.3)	0.29	0.8 (0.4, 1.3)	0.32
Posterior	1.0 (0.5, 1.9)	0.98	1.0 (0.5, 2.0)	0.98	1.0 (0.5, 2.0)	0.98
**Femoroacetabular BMLs** ^3^						
Anterior	0.9 (0.5, 1.8)	0.76	1.2 (0.6, 2.4)	0.63	1.2 (0.6, 2.5)	0.65
Central superolateral	2.3 (1.3, 3.9)	<0.01	2.1 (1.2, 3.7)	0.01	2.1 (1.2, 3.8)	0.01
Central inferomedial	0.6 (0.2, 2.1)	0.42	0.8 (0.2, 3.2)	0.74	0.7 (0.2, 3.0)	0.66
Posterior	1.8 (1.0, 3.5)	0.07	2.3 (1.1, 4.7)	0.03	2.3 (1.1, 4.9)	0.03

^1^Regression coefficient (β) (95% CI) where for every one unit increase in the exposure (occupational heavy lifting), there is an associated increase (+) or decrease (−) in femoral head cartilage volume (mm^3^) adjusted for age, gender, BMI, femoral head bone area and MRI centre in Model 1; ^2^odds ratio (OR) where for every one unit increase in the exposure (occupational heavy lifting), there is an associated increased risk (value >1) or decreased (value <1) risk for cartilage defects, adjusted for age, gender, BMI, femoral head cartilage volume and MRI centre in Model 1; ^3^odds ratio (OR) where for every one unit increase in the exposure (occupational heavy lifting), there is an associated increased risk (value >1) or decreased (value <1) risk for BMLs adjusted for age, gender, BMI and MRI centre in Model 1; ^4^Model 2: vigorous physical activity in past 7 days and heavy domestic chores in past 7 days added to Model 1 multivariate equation. CI, confidence interval; BMLs, bone marrow lesions; BMI, body mass index; MRI, magnetic resonance imaging.

## Discussion

Occupational exposure to heavy lifting and stair climbing are associated with hip structural abnormalities including cartilage defects and BML in the central superolateral region of the joint. If confirmed by longitudinal data, such associations may help to explain how occupational activities affect the hip joint and may identify new targets for the prevention of hip OA.

A previous systematic review reported a consistent association between heavy lifting and the risk of hip OA (OR ranging from 1.97 to 8.5) [2] but the studies selected advanced OA defined by either the need for total hip arthroplasty (THA) or radiographic disease. For instance, high exposure to heavy lifting when aged 30 to 49 years was reported for men awaiting THA for OA [14]. Similarly, another study found that THA for OA was more common for men lifting 10 kg or more before the age of 30 years [5]. Neither of these previous studies examined structural outcomes, although another study found that 60- to 75-year-old men with lifetime exposure to heavy lifting demonstrated severe joint space narrowing measured on intravenous urogram [3]. In the current study, heavy lifting when aged 18 to 30 years was associated with an increased risk of BMLs in the central superolateral region of the femoroacetabulum, while the persistence score for heavy lifting score was associated with an increased risk of cartilage defects in the central superolateral region of the femoral head. Taken together, these results infer that occupational heavy lifting is associated with deleterious structural changes in the hip joint of adults with no diagnosed hip OA.

This study has also demonstrated that stair climbing aged 18 to 30 years, as well the persistence score were associated with increased risk of cartilage defects in the central superolateral region of the femoral head and BMLs in the central superolateral and posterior femoroacetabulum. Although the current study has not examined people with hip OA, previously studies have found equivocal associations between stair climbing and hip OA, defined by radiographic disease or the requirement for THA. In a systematic review, three of five studies found a significantly increased risk of hip OA with stair climbing [2]. For instance, a case-control study found that males awaiting THA were more likely to have been exposed to stair climbing [5]. Other studies have demonstrated similarly positive associations, although they did not reach statistical significance [3,6,7]. This may be due to the heterogeneity of variables examined, such as climbing >30 flights of stairs a day in people awaiting THA [6] or alternate cutoffs of climbing >15 flights of stairs a day in a mixed group of people who had either received or were awaiting THA, or had severe radiographic OA [7]. Although requiring further investigation, the current study demonstrates that occupational exposure to stair climbing of at least 30 flights of stairs a day for at least one year is associated with structural abnormalities in the hip joint that may signify early hip OA.

This study failed to demonstrate any significant associations between occupational activities and femoral head cartilage volume. Recently, we have demonstrated that compared to people with hip OA, people without diagnosed hip OA have significantly greater cartilage volume [15]. Nevertheless, even among people without hip OA, the presence of a cartilage defect at the hip is associated with significantly less femoral head cartilage volume [15]. These data suggest a continuum from asymptomatic disease with structural damage, through to well-established hip OA. Despite no occupational activity being associated with reduced femoral head cartilage volume, the associations between cartilage defects and heavy lifting or stair climbing is likely to signify early abnormalities in cartilage integrity.

Although previous studies have focussed on association between an occupational exposure and the prevalence of hip OA, they have not explored how structural abnormalities in the joint may mediate the trajectory toward a diseased state. Our study is the first to demonstrate an association between occupational activities and early changes of hip OA but the mechanism accounting for the associations of structural hip abnormalities with stair climbing and heavy lifting is unclear. Load increases to five to seven times body weight when climbing stairs and three times body weight when heavy lifting [14,16]. Such load increments may overburden articular structures and cause early deleterious changes such as cartilage defects and BMLs in axial joints, such as the hip. Another mechanism may be that with repetitive exposure to activities such as stair climbing and heavy lifting, bone geometry is modified. There is evidence that hip bone geometry may increase the risk of radiographic hip OA [17]. It is also plausible that heavy occupational loads applied to a hip joint with pre-existing subtle abnormalities in shape (for example femoroacetabular impingement) may cause accelerated structural damage. Moreover, since significant results from this study were consistently found in the central superolateral and posterior, rather than the central inferomedial and anterior regions of the hip joint, it may be that heavy lifting and stair climbing exert a location-specific pathology at the hip. Finally, it is important to acknowledge that these results were observed for a cohort of adults who had survived on average, to retirement age with no diagnosis of hip OA. It is possible that people in this study had some extraneous protection from developing overt clinical hip joint failure in response to such occupational activities.

This study has several limitations. Participants were asked to nominate their occupational exposure when aged 18 to 30 years and in the past 10 years, with the potential for recall bias. Nevertheless, participants would not have been aware of their MRI structural abnormalities, mitigating any influence that recall bias pertaining to occupational exposures may have had on the primary endpoint of the study (that is structural damage). Moreover, the persistence score was derived from these two time points. It is assumed that people who nominated occupational exposure when aged 18 to 30 and in the previous 10 years had maintained a similar pattern of occupational exposure. This is likely to be the case as there was a strong correlation between the two time points for both heavy lifting (r = 0.81, *P* <0.001) and stair climbing (r = 0.85, *P* <0.001). Since the aim of this study was to examine how occupational activity was associated with hip structural abnormalities, we do not present analyses related to occupational activities in the previous 10 years because occupational activities at this time point may have been influenced by the structural changes at the hip. Moreover, people who had participated in occupational stair climbing or heavy lifting at only one time point were considered to represent one group (that is grade 1). This group was devised to identify people who had performed at least some, albeit inconsistent occupational exposure to the variable of interest. It is likely that this conservative approach of grouping these participants together may have resulted in non-differential misclassification and thus reduced the likelihood of showing any significant effect. Moreover, although the majority of subjects in this study were female, a greater proportion of males rather than females had participated in stair climbing (11.7% versus 1.7%) and heavy lifting (37.2% versus 22.7%) at both time points (both *P* <0.01). These data indicate that males were more likely to have had greater persistence scores to more laborious occupations and larger studies may be required to determine whether women with laborious occupations have similar associations. Whilst we have taken occupational, physical and domestic activity into account, it is likely that we have not captured all relevant factors and so the potential for unmeasured confounding accounting for some of these relationships remain. Additionally, people with no significant history of hip disease or symptoms were recruited and they did not have radiographs performed in this study. Although some participants may have had early radiographic OA, they did not have sufficient symptoms to seek medical diagnosis or intervention. In this study, we have measured the presence or absence of a BML and have not measured its size; future studies would benefit from a quantitative measure of BML size. Finally, it has been notoriously difficult in epidemiological studies to assess structural changes at the hip joint using MRI. Our division of the anterior, central and posterior regions was adapted from methods used in previously published works with smaller sample sizes [10,11] but these previous works provided no prevalence data of regional structural abnormalities for comparative purposes. Our approach has provided the first evidence for an association between occupational exposure and regional structural abnormalities on hip MRI.

## Conclusions

We have demonstrated that occupational exposure to heavy lifting and stair climbing are associated with structural hip abnormalities (cartilage defects and BMLs) in community-based adults without a diagnosis of hip OA. If confirmed by longitudinal follow-up, these results suggest that stair climbing and heavy lifting have an important role in the pathogenesis of early structural changes that may herald clinical hip OA.

## Abbreviations

BMI: body mass index
BML: bone marrow lesion
CI: confidence interval
CV: coefficient of variation
ICC: intraclass correlation coefficient
MCCS: Melbourne Collaborative Cohort Study
MRI: magnetic resonance imaging
OA: osteoarthritis
OR: odds ratio
PASE: Physical Activity Scale for the Elderly
THA: total hip arthroplasty
WOMAC: Western Ontario and McMasters Universities Arthritis Index
